# A growth model for directed complex networks with power-law shape in the out-degree distribution

**DOI:** 10.1038/srep07670

**Published:** 2015-01-08

**Authors:** J. Esquivel-Gómez, E. Stevens-Navarro, U. Pineda-Rico, J. Acosta-Elias

**Affiliations:** 1 Facultad de Ciencias, Universidad Autónoma de San Luis Potosí (UASLP), México; 2 Instituto de Investigación en Comunicación Óptica, Universidad Autónoma de San Luis Potosí (UASLP), México

## Abstract

Many growth models have been published to model the behavior of real complex networks. These models are able to reproduce several of the topological properties of such networks. However, in most of these growth models, the number of outgoing links (*i.e.*, out-degree) of nodes added to the network is constant, that is all nodes in the network are born with the same number of outgoing links. In other models, the resultant out-degree distribution decays as a poisson or an exponential distribution. However, it has been found that in real complex networks, the out-degree distribution decays as a power-law. In order to obtain out-degree distribution with power-law behavior some models have been proposed. This work introduces a new model that allows to obtain out-degree distributions that decay as a power-law with an exponent in the range from 0 to 1.

In the literature, there are many growth models for complex networks (*CN*) that reproduce some topological properties of real systems^1^. However, in most growth models it is assumed that all nodes are born with the same amount of outgoing links (*i.e.*, their out-degree is a constant), as in the model proposed by Barabási-Albert^2^. In other models, such as the one proposed by Dorogovtsev *et.al*^3^ and the one proposed by Krapivsky and Redner^4^, the out-degree distribution decays as an exponential or a poisson distribution, respectively. However, these results differ from the out-degree behavior of several real *CN*. For example, in metabolic networks^5^, the Internet^6^, and *WWW*^7^ the out-degree decays as a power-law. Therefore Dorogovtsev *et.al.*^8^ and Bollobás *et.al.*^9^ developed two models that are able to produce out-degree distributions that decay as a power-law with exponent 
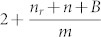
 and 
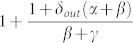
 respectively, that is, in both models the exponent is greater than 2. It is important to mention that for the average out-degree to be finite in the infinite system size limit the exponent must be larger than 2. Since any exponent smaller or equal to 2 results in a distribution with diverging first moment, *i.e.* where the average out-degree of nodes is infinite when *N* → ∞.

In the present work, we propose a simple growth model for directed *CN* which is able to generate out-degree distributions that decay as a power-law with exponent 0 < *γ_out_* < 1. In the proposed model, the growth of the network is done by adding nodes one at a time. At the beginning, only the node *n*_0_ exists in the network and its out-degree is 0. Then we consider that the out-degree of any new node *n_new_* added to the network is determined as follows:

- with probability *p* where 0 < *p* < 1, *n_new_* copies the out-degree of a randomly selected node from the network. It is important to note that as the quantity *Q_s_* of nodes with out-degree *s* increases, the probability that node *n_new_* has out-degree *s* also increases to 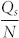, where *N* is the total number of nodes in the network.
- with complementary probability 1 − *p*, *n_new_* randomly selects an out-degree uniformly distributed from 0 to *N*. That is, node *n_new_* has out-degree 0, 1, 2, … *N*. It is important to note that this rule produces unrealistic out-degree of the new node almost all the times it is applied. That is, new nodes may have out-degree of the order *N*.

By applying the previous considerations and using the continuum method^10^, we can write the following differential equation:


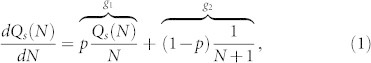


that describes the variation of the quantity *Q_s_* of nodes with out-degree *s* with respect to the total number *N* of nodes in the network. The term *g*_1_ accounts for the situation that a new node copies the out-degree of a randomly selected node in the network. The term *g*_2_ describes the random selection of out-degree for a new node.

Eq. 1 can be written in the standard form for a linear differential equation as follows:


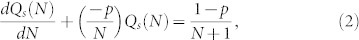


multiplying by the integrating factor 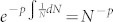, we obtain


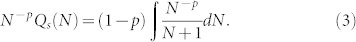


Since to the integral of Eq. 3 is not elementary, the solution retrieved is in terms of the Hypergeometrical Function _2_*F*_1_ as follows:


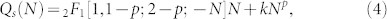


where *k* is a constant. To obtain the out-degree distribution *Q_s_*(*N*), we solve Eq. 4 for *s* = 1, *s* = 2, and so on as follows:

•  for *Q*_1_(*N*), we need to consider the initial condition


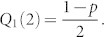
This initial condition is due to the fact that at the beginning, the network is formed only by node *n*_0_ with no outgoing links, that is *N* = 1. For this case the quantity *Q*_1_(1) of nodes with out-degree *s* = 1 is zero (*Q*_1_(1) = 0). When the node *n*_1_ is added (*N* = 2), the probability for node *n*_1_ to have out-degree *s* = 1 is 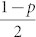. Solving Eq. 4 for the initial condition 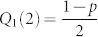, we obtain:


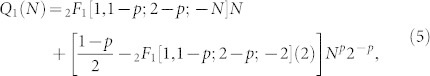


•  for *Q*_2_(*N*), we need to consider the initial condition


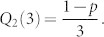
This initial condition is due to the fact that, before adding node *n*_2_, only nodes *n*_0_ and *n*_1_ are in the network (*N* = 2) and any of them has *s* ≥ 2, therefore *Q*_2_(2) = 0. When node *n*_2_ is added (*N* = 3), the probability that node *n*_2_ has out-degree *s* = 2 is 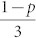. Solving Eq. 4 for the initial condition 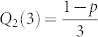, we obtain:


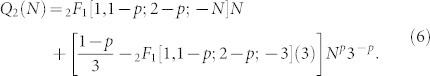


From the previous results in Eqs. 5 and 6, we can deduce that:


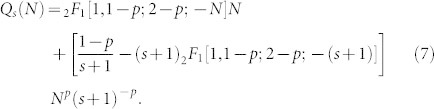


Normalizing Eq. 7 we obtain


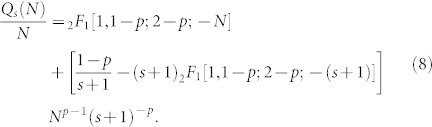


Eq. 8, shows that the exponent *γ_out_* of the out-degree distribution obtained with the proposed model is only determined by the probability *p*. That is, the out-degree distribution obtained decays as a power-law


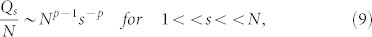


with exponent *γ_out_* = *p*.

On the other hand, we can deduce that as a consequence of the random out-degree selection by new nodes with probability 1 − *p* (second rule of the proposed model), the average out-degree of the nodes grows with the network size. To validate this hypothesis, we analytically calculate the average out-degree 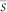 using the following differential equation:


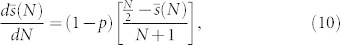


that describes the increment of the average out-degree 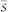 with respect to the total number *N* of nodes in the network. On the right-hand side of Eq. 10, the term 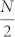 describes the mean of the random out-degree uniformly selected from 0 to *N* by a new node. Thus, the term 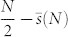 describes the increment of 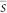.

Eq. 10 can be written in the standard form for a linear differential equation as follows:


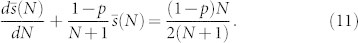


Solving Eq. 11 we obtain


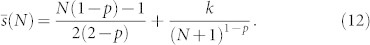


As the total number of nodes in the network increases (*N* ≫ 1), we can approximate Eq. 12 as follows:


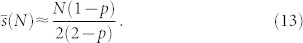


From Eq. 13 we can see that effectively 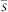 grows proportionally to the network size, that is, in the proposed model the average out-degree of nodes is infinite when *N* → ∞.

In order to validate the analytical solutions for the out-degree distribution (Eq. 8) and average out-degree (Eq. 13) of the proposed model, we performed four numerical simulations using *p* = 0.1, *p* = 0.3, *p* = 0.6, and *p* = 0.9. In each simulation, we considered the growth of a directed network from 1 to 10^4^ nodes. Figure 1a shows that the results of the numerical simulations and the analytical prediction (Eq. 8) for the out-degree distribution fit appropriately. On the other hand, we measure the average out-degree 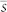 in each simulation for different network sizes. Figure 1b shows that the average out-degree retrieved from the simulations and the analytical prediction (Eq. 13) fit also appropriately. That is, in the proposed model the average out-degree 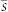 grows linearly with *N* for any value of 0 < *p* < 1 as stated by Eq. 13 and consequently the average out-degree of nodes is infinite when *N* → ∞. This contrasts with some large networks that are sparse where the number of edges is much smaller than the maximum possible and the average out-degree increases slowly as the network grows^11^.

The topological properties of real *CN* seems to be the result of a set of local processes. We consider that the proposed model in this work can contribute to develop new growth models for directed *CN* which consider local processes that shape the out-degree of the nodes and, therefore, produce better predictions of the behavior of real *CN* and thus increases the understanding of these systems.

## Author Contributions

J.E.G., E.S.N., U.P.N. and J.A.E. designed, performed the research and wrote the manuscript.

## Figures and Tables

**Figure 1:**
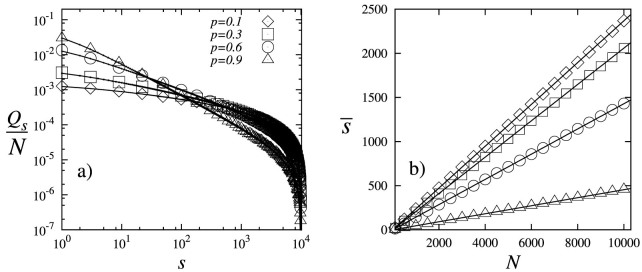
(a) Comparison of the out-degree distribution (symbols) retrieved from the simulations and the analytical predictions (lines). (b) Comparison of the Average out-degree 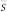 retrieved from the simulations for different network sizes and the analytical predictions (lines).
